# The challenges of global bioethics

**DOI:** 10.1080/11287462.2021.2011008

**Published:** 2022-02-04

**Authors:** Henk ten Have

**Affiliations:** Center for Healthcare Ethics, Duquesne University, Pittsburgh, USA

**Keywords:** Care-based ethics, Covid-19 pandemic, global bioethics, human rights, vulnerability

## Abstract

The Covid-19 pandemic is associated with an increase in ethics publications and an upsurge of interest in global bioethics. This commentary argues that global bioethics is broader than international bioethics, as defined by Macklin, because the nature of moral problems is determined by processes and practices of globalization, and because a broader theoretical perspective is required. Such perspective acknowledges the connectedness and relationality of human beings, as assumed in the care-based feminist bioethics defended by Tong. The commentary finally claims that a rights-based approach is not opposed to but reinforces a care-based global bioethics.

The Covid-19 pandemic caused a boom of ethics publications. A PubMed search with keywords “ethics & Covid-19” shows 2525 publications in 2020 (up from 3 in 2019) and 1729 in the first half of 2021. These publications cover a wide range of issues: crisis standards of care, expedited research, duty to care, triage in intensive treatment, prioritization of vaccination. The majority of studies are concerned with moral dimensions of treatment (1419 in 2020; 802 in 2021). It is interesting, however, that several publications engage in self-reflection, arguing that bioethics is insufficiently addressing issues of community, the common good, solidarity and fairness, and that post-Covid bioethics should have a global perspective (Ravitsky, 2020). The phenomenon of a pandemic reactivates the notion of global bioethics. It is helpful to consider why this happens since it clarifies how global bioethics should be interpreted and why Ruth Macklin’s definition is too limited. In her view, global bioethics is the study of “the ethical aspects of relations between and among nations or regions of the world”. Specific ethical problems arise in international relations, and Macklin gives several examples. However, what makes global bioethics different from international bioethics is the context of globalization which not only intensifies these relations but transforms them with pervasive implications for the lives of people, wherever they are located. More is needed than the exploration of the relations between nations and regions, because moral challenges arise due to the varying impact of processes of globalization on citizens across the world. Of course, solidarity, cooperation and diplomacy are crucial but not merely between and among nations. The WHO, given a central place in Macklin’s article, has a role in promoting global bioethics, but many other international organizations can make essential contributions (e.g., FAO, UNESCO and WIPO). At the same time, the impact of such organizations is limited, and global bioethical perspectives are often advanced at regional, national and local levels by NGOs, civil initiatives, ethics committees and individual experts.

Restricting global bioethics to international bioethics is inadequate for two reasons. One is the nature of contemporary ethical challenges. As the Covid pandemic illustrates these challenges have a specific character as global phenomena. Even if they originate in a specific locality (e.g. the SARS-CoV-2 virus), they cannot be contained locally but spread around the globe. They are not local, national and regional but global threats, requiring global responses. Other challenges such as climate change, poverty and loss of biodiversity do not emerge in a specific place but are disproportionately affecting some countries and regions which can only cope with them through global cooperation and action. These problems are fundamentally different from the ones that have confronted bioethics thus far, not only because of their global nature but also because they are intimately connected to processes and practices of globalization that have characterized international relations. To address these problems demand critical analysis of these processes and their inherent moral values and perspectives. This brings us to the other reason to advocate a broader bioethical approach. The dominant discourse of bioethics is likewise concerned with international relations, assuming that its methodology and ethics frameworks apply to all nations and regions. It is no longer, as Macklin rightly observes, concerned with ethical issues arising in a particular country or the developing world but has a global outreach. Nonetheless, recognition that nowadays bioethics is facing another type of problems due to globalization does not imply the recognition that a different methodology with a wider set of theoretical perspectives is requested. The need for a broader approach is manifested in current publications that question the dominant discourse of bioethics during the pandemic with its emphasis on individual autonomy, and neglect of solidarity, vulnerability and inequity (Fins, 2020; Ho & Dascalu, 2020). Global bioethics differs from bioethics since as a response to a specific kind of problem it has a different moral orientation, and this is the issue addressed by Rosemary Tong.

The perspective of global bioethics is determined by two dimensions of “global” discerned by Potter (and quoted in Tong’s contribution): it has a worldwide scope, and it is broad and encompassing. Both dimensions are illustrated in the Covid-19 pandemic: the viral threat is a planetary phenomenon, and the pandemic is not just a medical and virological crisis but also a social, environmental and political one. Global bioethics has been defined as “The study of global ethical problems related to health, healthcare, health science and research, and health technologies and policies, and the activities, practices and policies to influence and resolve these global problems” (Ten Have, 2016, p. 243). But this definition only specifies the first dimension; it describes the field of work rather than the broad moral orientation. It does not articulate that global bioethics is inspired by the ideals of cosmopolitanism: the unity of humanity, solidarity, equality, openness to differences, and focus on what human beings have in common. These ideals consider each human being as a citizen of his or her own community or state as well as at the same time as citizen of the world. In the first, they are born; they share a common origin, language and customs with co-citizens. In the second, they participate because they belong to humanity; all human beings share the same dignity and equality. Being citizen of the world liberates the individual from captivity in categories such as culture, tradition and community, but also gender and race. Cosmopolitanism acknowledges that human beings are connected to other beings and the environing world. Potter employs the image of bridge to refer to connectedness as the familiar experience of globalization. This anthropological experience (the “togetherness” frequently highlighted during the pandemic) should be the starting point for ethical reflections that take human relationality more seriously. If humans not merely interact with each other but belong together and are mutually dependent, taking responsibility and shaping their lives in sharing the world, relationships with others define who a person is. Individual autonomy should then be redefined as a relational concept. Community, mutual support, social responsibility, cooperation and solidarity should have a significant role in inclusive and comprehensive bioethical discourse. Furthermore, being situated in a web of connections is a precarious experience. Because their bodies position them in the world, human beings are exposed to the world and other persons, necessarily implying vulnerability. I assume that this is the same ethical perspective developed by Tong. She argues that care has priority over justice; without care, human rights will not be respected. I agree but at the same time wonder whether the opposition between rights-based and care-based approaches is not too strong. While it is true that rights are often associated with autonomous and independent individuals, a relational interpretation of rights emphasizes the connectedness of human beings and directs attention to social, economic and political conditions that are often oppressive and unjust. Rights can amplify the significance of care, for example stipulating that health research does not justify the exploitation of some citizens, taking advantage of poverty, lack of development or social misery to advance knowledge. In a globalized world, issues such as inequality, exclusion, exploitation and vulnerability continue to arise, directing attention to the normative settings of the context itself. Rights are powerful instruments to criticize and reshape this context. They are furthermore important since many bioethical problems are identified and addressed through “globalization from below,” i.e. through grassroots organizations and local activists as a third sector between state and market. Human rights and global principles are used to challenge local practices while local knowledge feeds back into the global framework.

Policy-making in healthcare is often driven by concerns for vulnerable populations in need of care and protection. As is obvious in humanitarian aid, for example in disaster relief, the ethical drive to assist is so strong and compelling that it can hardly be criticized; at the same time, it directs our focus on immediate care and relief for individual victims so that we tend to forget that other dimensions are equally important. One dimension is the social context which is often unjust. Another dimension is the perspective of the recipient which is often absent and silent. Bringing in the discourse of rights makes explicit that people should not be regarded as needed victims but as citizens of the world with the same claims and rights as everyone else. Rights dignify rather than victimize; they make people equal and more powerful, and increase the impact of care-based global bioethics.
